# The molecular revolution in fungal diagnostics: bridging gaps across clinical, agricultural, and environmental mycology

**DOI:** 10.1080/21501203.2025.2569931

**Published:** 2025-12-03

**Authors:** Youssuf A. Gherbawy, Helal Al-Harthi, Eman El-Dawy, Mahmoud Gaber, Ioan Pet, Linqi Wang, Pengjie Hu, Mohamed Hussein

**Affiliations:** a Botany and Microbiology Department, Faculty of Science, South Valley University, Qena, Egypt; b Applied and Environmental Microbiology Center, South Valley University, Qena, Egypt; c Biology Department, Turabah University College, Taif University, Taif, Saudi Arabia; d Department of Plant Pathology, Faculty of Agriculture, Alexandria University, Alexandria, Egypt; e Biotechnology Department, Faculty of Bioengineering and Animal Resources, University of Life Sciences “King Mihai I” from Timisoara, Timisoara, Romania; f State Key Laboratory of Mycology, Institute of Microbiology, Chinese Academy of Sciences, Beijing, China; g University of Chinese Academy of Sciences, Beijing, China

**Keywords:** Molecular diagnostics, fungal identification, DNA barcoding, next-generation sequencing, cryptic species

## Abstract

Fungi play essential roles in human health, agriculture, and ecosystems, yet their diversity has long been underestimated due to reliance on morphology and culture. Molecular innovations—DNA barcoding, multilocus and whole-genome sequencing, NGS, and rapid nucleic acid diagnostics (qPCR, LAMP, CRISPR/Cas)—have revolutionized fungal taxonomy and detection. The ITS region now serves as a universal barcode, often complemented by other loci or genomic data. High-throughput and portable tools enable near real-time identification, supported by AI-driven bioinformatics for species recognition and resistance prediction. Despite progress, challenges remain in database accuracy, primer design, and standardization. Integrating molecular taxonomy, AI, and global collaboration promises scalable, reliable frameworks for fungal surveillance and control across clinical, agricultural, and environmental domains.

## GRAPHICAL ABSTRACT

**Figure:**
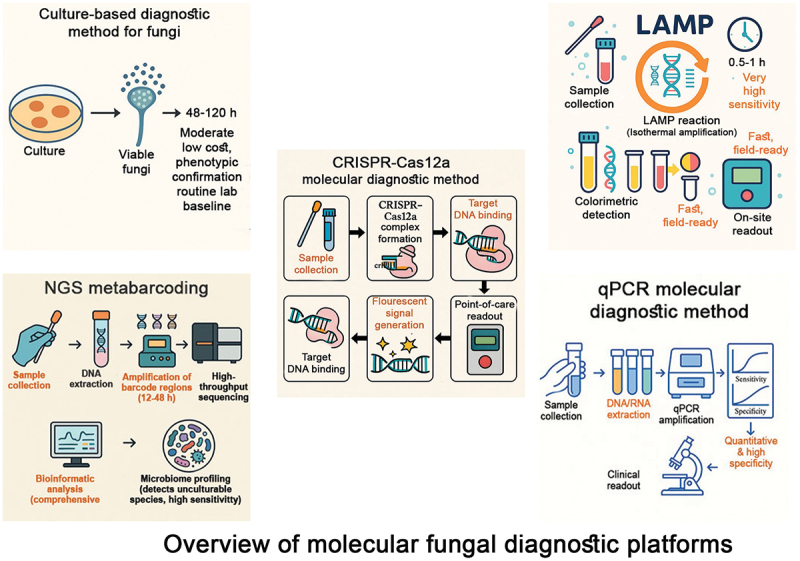

## 1. Introduction

Fungi represent a kingdom with remarkable diversity, estimated at around 2.2 to 3.8 million species, many of which have yet to be discovered and formally described (Hawksworth and Lücking 2017). Given their widespread presence and important ecological functions, identifying and classifying fungal species accurately is critical. Recent molecular methods, including polymerase chain reaction (PCR) and high-throughput sequencing, have greatly enhanced the accuracy of fungal taxonomy and diagnostic processes. By surpassing the limitations of traditional morphology-based techniques, these methods have provided deeper understanding of fungal diversity, ecological interactions, and pathogenic traits (Gherbawy and Voigt 2010; Hariharan and Prasannath 2021).

### 1.1. The ubiquity and impact of fungi

#### 1.1.1. Clinical perspective

Fungal pathogens are increasingly recognised as a significant threat to global health, particularly among immunocompromised populations, including cancer patients, organ transplant recipients, and individuals living with HIV/AIDS. Invasive fungal infections (IFIs), most notably those caused by *Candida*, *Aspergillus*, and *Cryptococcus* species, are associated with considerable morbidity and mortality, with rates often exceeding 50% in high-risk groups (Brown et al. 2012). The emergence of multidrug-resistant fungi—most prominently *Candida auris*, which has been designated a “critical priority” by the World Health Organization—highlights the urgent need for improved diagnostic modalities and enhanced epidemiological surveillance (World Health Organization 2022). Resistance mechanisms, such as ERG11 gene mutations and efflux pump overexpression, are exacerbated by the widespread use of azoles in both medical and agricultural settings, posing substantial therapeutic challenges (Perlin et al. 2017).

#### 1.1.2. Agricultural perspective

Fungal diseases are among the leading causes of crop losses worldwide, with estimates attributing up to 20% of annual yield reductions to fungal pathogens. This represents a significant threat to food security and economic stability (Fisher et al. 2012). Certain species, such as *Fusarium graminearum* (the etiological agent of wheat head blight) and *Aspergillus flavus* (a producer of aflatoxins), are particularly problematic, contaminating staple crops like maize and rice with carcinogenic mycotoxins. The effects of climate change have further exacerbated these issues by expanding the ecological ranges of toxigenic fungi (Faraj et al. 1991). The economic impact is considerable; for example, aflatoxin-related trade losses alone are estimated to exceed $1.2 billion annually (Mitchell et al. 2016).

#### 1.1.3. Environmental perspective

Within ecological systems, fungi play a pivotal role, often serving as keystone species. They facilitate the decomposition of organic matter, enable nutrient cycling, and contribute to carbon sequestration (Crowther et al. 2012). Mycorrhizal associations enhance plant resilience to environmental stressors, while saprotrophic fungi are instrumental in maintaining soil fertility. Notably, fluctuations in fungal communities can act as sensitive bioindicators of disturbances such as pollution, land use changes, or climate variability (Warnasuriya et al. 2023). Recent methodological advances, including environmental DNA (eDNA) metabarcoding, have uncovered remarkable fungal diversity—even in extreme environments like Arctic permafrost and deep-sea hydrothermal vents (Warnasuriya et al. 2023).

### 1.2. Challenges with traditional methods

#### 1.2.1. Historical approaches to fungal identification

Before molecular techniques took over, fungal identification mostly relied on classical morphology and culture-based approaches. To break it down:

(1) Morphological analysis: Researchers assessed visible characteristics like colony colour, texture, and how fast the fungus grew. On the microscopic level, they’d examine details such as spore shape and the structure of conidiophores to distinguish genera such as *Aspergillus* and *Penicillium* (Gherbawy and Voigt 2010; Visagie et al. 2014; Alsohaili and Bani-Hasan 2018).

(2) Substrate-based sampling: Fungi were collected from various substrates—soil, wood, plant debris, and so on. In tougher cases, moist chambers were used to induce sporulation, making microscopic examination possible (Gherbawy and Voigt 2010; Overy et al. 2019).

(3) Culture media: Standard practice involved growing fungal isolates on artificial media like potato dextrose agar, observing colony morphology, and sporulation patterns. This method remains central for identifying plant pathogens and environmental isolates.

(4) Taxonomic keys and reference works: Identification was performed by comparing observed morphological features with authoritative descriptions found in works such as Ainsworth & Bisby’s Dictionary of the Fungi (Hawksworth et al. 1995; Kirk et al. 2001; Gherbawy and Voigt 2010).

(5) Limitation: misannotation and cryptic speciation: It is estimated that 10%–20% of fungal sequences deposited in major repositories such as GenBank are misannotated, with the issue being especially acute in taxa characterised by cryptic speciation, such as *Aspergillus* section *Nigri* and the *Candida parapsilosis* complex (Balajee et al. 2009; Nilsson et al. 2019). This persistent problem reflects challenges in reconciling molecular discoveries with traditional nomenclatural frameworks, as well as the practical difficulties of linking genetic data to morphological type specimens.

#### 1.2.2. Contemporary limitations

While these traditional approaches provided the foundation for mycological research, several critical limitations have become apparent:

(1) Cryptic speciation: Many morphologically similar species, particularly within groups such as *Aspergillus* section *Nigri*, are indistinguishable without molecular data. This leads to frequent misidentification of toxigenic strains (Balajee et al. 2009; Gherbawy and Voigt 2010).

(2) Subjectivity and reliance on expertise: The accuracy of identification is heavily dependent on the skill of the taxonomist. Studies indicate intra- and inter-observer variability can exceed 30% in both clinical and environmental contexts (Balajee et al. 2009).

(3) Culturability limitations: A significant majority of environmental fungi—up to 95%—cannot be cultured using standard laboratory methods, resulting in incomplete assessments of fungal diversity and potentially missing important pathogens (Wijayawardene et al. 2021).

(4) Time and resource constraints: Culture-based diagnostics are inherently slow, often requiring three to 14 days for reliable identification. This delays interventions in cases of invasive infections or crop diseases (Cornely et al. 2014).

(5) Need for molecular, culture-independent methods: These challenges collectively demonstrate the pressing need for molecular, culture-independent methodologies. Such approaches are essential for resolving cryptic species complexes, overcoming the limitations of unculturable taxa, and addressing the growing problem of antifungal resistance (Gherbawy and Voigt 2010; Wijayawardene et al. 2021).

#### 1.2.3. Nomenclatural implications for molecular diagnostics

While these challenges set the stage for the adoption of molecular, culture-independent methodologies, it is critical to recognise an additional foundational barrier: the formal fungal naming system itself.

(1) The International Code of Nomenclature (ICN) requirement: According to the ICN, validly published fungal species must be anchored to a physical, morphologically defined type specimen (Gherbawy and Voigt 2010; Nilsson et al. 2019). This requirement enforces taxonomic stability by linking names to tangible reference material.

(2) Constraint in the molecular era: This foundational requirement creates a significant barrier in the era of DNA-based diagnostics, where many cryptic or unculturable species are only diagnosable via sequence data (Nilsson et al. 2019; Kõljalg et al. 2020). These unnameable lineages, commonly referred to as “dark taxa,” evade formal nomenclatural recognition despite increasing evidence of their ecological and clinical significance.

(3) Consequences for taxonomy and databases: As a result, genetically coherent but morphologically indistinguishable or unculturable lineages often lack valid names or receive ambiguous assignments in public repositories, contributing to ongoing taxonomic ambiguities and high rates of misannotation (Balajee et al. 2009; Nilsson et al. 2019). Community databases such as UNITE (2020, https://unite.ut.ee/) address this gap by clustering sequences into operational units labelled with Taxon Hypothesis (TH) identifiers. These stable TH IDs serve as pragmatic proxies for traditional taxon concepts, facilitating communication and reproducibility within molecular ecology and diagnostics, even for taxa lacking formal names.

(4) The challenge of integrating molecular and classical typification: Addressing the disconnect between classical typification (requiring physical specimens) and molecular species concepts is now a central challenge for achieving reliable, comprehensive fungal taxonomy in the age of genomics (Nilsson et al. 2019; Kõljalg et al. 2020). Proposed reforms to the ICN—including those under discussion at the 2024 International Botanical Congress in Madrid—seek to allow “sequence-based types” as valid nomenclatural types. Such reforms would enable TH units (and other rigorously defined molecular clusters) to be formally incorporated into the taxonomy with official names anchored by curated sequence data rather than physical specimens.

Operationally, this could transform TH identifiers from informal database constructs into recognised taxon names, harmonising molecular ecological data with formal nomenclature and resolving longstanding ambiguities associated with dark taxa. This integration would foster greater interoperability between curated molecular databases and global taxonomic frameworks, leading to more accurate fungal diagnostics, improved surveillance, and clearer communication across clinical, agricultural, and environmental mycology domains.

### 1.3. The molecular revolution

The integration of DNA-based technologies has dramatically reshaped the field of fungal taxonomy and diagnostics. The internal transcribed spacer (ITS) region is now firmly established as the standard fungal barcode, providing reliable species-level identification across diverse taxa (Schoch et al. 2012). For more nuanced cases, where cryptic species pose a challenge, multilocus sequencing—utilising markers such as TEF1-α and β-tubulin—has become routine (O’Donnell et al. 2015). Next-generation sequencing (NGS) and whole-genome sequencing (WGS) now enable differentiation at the strain level, facilitate outbreak investigations, and support resistance profiling (Lockhart et al. 2017). The advent of portable sequencing devices allows for on-site diagnostics, while curated databases like UNITE and MycoBank, coupled with AI-driven phylogenomic approaches, have markedly increased both the accuracy and scalability of analysis (Nilsson et al. 2019). Collectively, these advancements are bridging clinical, agricultural, and environmental diagnostics, providing rapid and precise insights into fungal biodiversity and pathogenic threats.

### 1.4. Rationale for this review

Despite considerable progress, several challenges remain—most notably in distinguishing cryptic species, monitoring antifungal resistance, and effectively assessing fungal diversity across sectors. Many existing reviews are limited by their focus on specific methodologies or single domains, which has resulted in a fragmented understanding of the field. This review aims to synthesise both established and emerging molecular techniques, critically evaluate their sector-specific applications, and discuss ongoing challenges as well as future directions. By addressing these aspects, the review seeks to inform researchers, clinicians, and policymakers about both the transformative potential and the limitations of molecular approaches, ultimately supporting the advancement of evidence-based research and policy development.

## 2. Core molecular techniques and innovations

The advent of molecular technologies has transformed the field of fungal diagnostics, offering notable improvements in accuracy, speed, and throughput (Table 1). These innovations are now integral to clinical, agricultural, and environmental mycology, facilitating solutions to challenges that traditional methods could not resolve, such as the identification of cryptic species, antifungal resistance profiling, and rapid pathogen detection.

**Table 1.** Comparison of traditional vs. molecular fungal diagnostic methods.

Method	Principle	Strengths	Limitations	Typical applications	References
Morphological identification	Microscopic/macroscopic analysis of fungal structures (hyphae, spores, etc.)	Low cost; widely accessible	Subjective; fails for cryptic species	Basic clinical/environmental surveys	Hawksworth et al. (1995); Hawksworth (2001)
Culture-based diagnostics	Growth on selective media (e.g., Sabouraud agar)	Enables antifungal testing; gold standard	Slow (2–5 days); misses unculturable fungi	Clinical/agricultural labs	Cornely et al. (2014); CLSI (2022)
DNA barcoding (ITS)	PCR amplification and sequencing of ITS region	Universal fungal barcode; high specificity	Limited cryptic species resolution	Biodiversity studies, clinical ID	Schoch et al. (2012); Nilsson et al. (2019)
Multilocus sequencing	Sequencing multiple gene regions (e.g., TEF1-α, β-tubulin)	Resolves cryptic species; higher resolution	Costly; requires expertise	Epidemiology, species complexes	Gherbawy and Voigt (2010); O’Donnell et al. (2015)
Next-generation sequencing (NGS)	High-throughput sequencing of entire communities	Detects unculturable species; community profiling	Expensive; bioinformatics challenges	Environmental metabarcoding, outbreak tracing	Fisher et al. (2012); Tedersoo et al. (2014)
Rapid molecular assays (qPCR, LAMP)	Targeted nucleic acid amplification and detection	Fast (< 2 h); field-deployable (LAMP)	Target-specific; limited multiplexing	Clinical diagnostics, field pathogen detection	Notomi et al. (2000); Tomlinson et al. (2005)
CRISPR-based diagnostics	CRISPR-Cas systems detect specific DNA/RNA sequences	Ultra-sensitive; portable; minimal equipment	Emerging tech; limited validation	Emerging clinical/agricultural diagnostics	Chen et al. (2018); Deivarajan et al. (2025)

### 2.1. DNA barcoding and multilocus sequencing

#### 2.1.1. ITS as the universal barcode

The Internal Transcribed Spacer (ITS) region is widely recognised as the primary marker for fungal identification. Its broad taxonomic range, significant interspecific variability, and straightforward amplification make it the standard in both research and diagnostic contexts (Schoch et al. 2012). As the most abundantly sequenced fungal locus in public databases, ITS underpins large-scale biodiversity studies and routine species identification (Nilsson et al. 2019).

Nevertheless, ITS does not always provide sufficient resolution to distinguish among closely related or cryptic fungal taxa. This limitation is particularly evident in genera such as *Aspergillus* and *Fusarium*, where morphological conservatism and overlapping ITS sequence variation pose challenges to precise species delimitation (Balajee et al. 2009). To overcome these obstacles, researchers have integrated additional protein-coding loci—most notably translation elongation factor 1-alpha (TEF1-α) and β-tubulin—into multilocus sequence typing (MLST) and multilocus sequence analysis (MLSA) frameworks (Gherbawy et al. 2015; Salehi et al. 2020). These markers offer complementary phylogenetic information, improving the accuracy of species identification and enhancing the reliability of taxonomic and epidemiological assessments. Table 2 provides an overview of the principal molecular markers currently employed in fungal identification, outlining their main applications, strengths, and limitations (Schoch et al. 2012; Gherbawy et al. 2015; Lockhart et al. 2017; Nilsson et al. 2019; Salehi et al. 2020).

**Table 2.** Comparative table summarising ITS1, ITS2, and 18S rRNA molecular markers.

Marker	Preferred applications	Taxonomic biases/scientific rationale	Case-specific insights & validation results	Validation approaches	Key references
ITS1	Soil, air, environmental samples; clinical mycobiome profiling	Favors Ascomycota; less plant DNA co-amplification; broad fungal diversity	Preferred in complex matrices such as soils due to reduced non-target amplification; mock community and field trials confirm higher richness for Ascomycota; lower Basidiomycota detection	In silico primer matching, spike-in (mock) communities, field validation in diverse samples	Schoch et al. (2012); Tedersoo et al. (2015); Nilsson et al. (2019)
ITS2	Plant tissues, crop diagnostics (e.g., *Fusarium graminearum*); some clinical contexts	Favors Basidiomycota, *Fusarium*, *Candida*; can capture more plant DNA	ITS2 outperforms ITS1 for *Fusarium graminearum* detection in wheat; validated in field and lab studies; higher specificity/sensitivity for certain pathogens	Comparative field/lab analysis, mock communities, infection studies, in silico tests	Ihrmark et al. (2012); Peay et al. (2016); Nilsson et al. (2019)
18S rRNA	Environmental DNA (water, sediments), broad eukaryote biodiversity surveys	Favors Chytridiomycota, Cryptomycota; lower taxonomic resolution for fungi; also captures protists and other microeukaryotes	Useful for broad eukaryote profiling; validated in mixed environmental samples; caution for fungi-specific studies	Broad environmental surveys, mock community tests, cross-validation with ITS	Elbrecht and Leese (2015)

#### 2.1.2. Multilocus sequencing: advancing taxonomic precision

When it comes to resolving cryptic fungal lineages, relying solely on the ITS region just does not provide sufficient resolution. To address this, researchers have incorporated protein-coding loci such as β-tubulin, calmodulin, and translation elongation factor 1-alpha (TEF1-α) into multilocus sequence typing (MLST) workflows. These loci offer increased phylogenetic signal due to their higher mutation rates and reduced homoplasy, enabling more accurate species delimitation (O’Donnell et al. 2015).

#### 2.1.3. *Case study: resolving* Aspergillus *section* Nigri *in agriculture*

Resolving species within the *Aspergillus* section *Nigri* complex continues to be a significant challenge in agricultural contexts, primarily because these fungi are morphologically indistinguishable yet differ in their mycotoxin production profiles. Conventional identification techniques simply do not cut it for such cryptic species. In response, Gherbawy et al. (2015) applied a multilocus sequence typing (MLST) approach, utilising β-tubulin and calmodulin gene sequencing in conjunction with species-specific primers. This method allowed for robust discrimination between species isolated from Egyptian grain samples. Notably, calmodulin-based primers proved particularly effective for differentiating *Aspergillus welwitschiae* from members of the closely related *A. niger* group.

**Figure 1.**
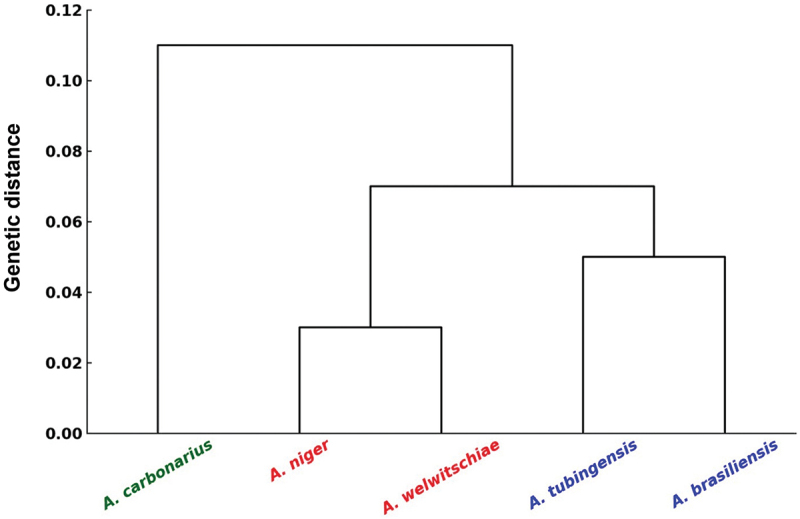
Phylogenetic analysis of *Aspergillus* section *Nigri*: multilocus approach. This dendrogram depicts genetic relationships among five key *Aspergillus* section *Nigri* species based on β-tubulin and calmodulin sequences. Distinct clustering (0.02–0.11 genetic distance) allows for accurate identification of cryptic, morphologically similar taxa relevant to food safety. The analysis follows protocols from Perrone et al. (2011), Gherbawy et al. (2015), and the taxonomy of Samson et al. (2014).

Building on this, Gherbawy et al. (2025) further refined the approach by combining β-tubulin and calmodulin markers with targeted PCR primers to characterise *Aspergillus* section *Nigri* species from grains in Upper Egypt. Their phylogenetic analyses (Figure 1) delineate clear genetic distinctions among key species such as *A. carbonarius*, *A.* *niger*, *A.* *welwitschiae*, *A.* *tubingensis*, and *A. brasiliensis*, underscoring the improved taxonomic resolution achieved through multilocus data.

This molecular approach not only advances our understanding of species boundaries within this economically important complex but also supports more accurate phylogenetic and epidemiological studies—a critical step for food safety surveillance and mycotoxin risk mitigation. Table 2 provides a concise summary of the principal molecular markers employed in fungal identification, detailing their respective applications, strengths, and limitations (Schoch et al. 2012; Gherbawy et al. 2015; Lockhart et al. 2017; Nilsson et al. 2019; Salehi et al. 2020).

### 2.2. High-throughput sequencing

#### 2.2.1. NGS for metagenomics and mycobiome analysis

Next-generation sequencing (NGS) has substantially advanced the study of fungal communities, providing unprecedented resolution and scope in both clinical and environmental contexts. The internal transcribed spacer (ITS) region serves as the standard fungal DNA barcode, widely employed for the detection and relative quantification of operational taxonomic units (OTUs) and amplicon sequence variants (ASVs). ITS1 is frequently favoured for its taxonomic precision, particularly in complex matrices such as soils, host tissues, or clinical samples (Schoch et al. 2014).

(1) Primer selection: ITS1 vs ITS2: The selection of ITS1 or ITS2 primers is not straightforward; it depends on the nature of the sample and the specific fungal groups targeted. ITS1 is generally favoured for soil and mixed environmental samples because it effectively amplifies a broad range of Ascomycota and tends to avoid excessive co-amplification of plant DNA, making it well-suited for profiling complex microbiomes (Tedersoo et al. 2015).

However, ITS2 may be a better choice under certain circumstances. For instance, when detecting *Fusarium graminearum* in Ethiopian wheat fields or other plant tissues, ITS2 primers have shown greater specificity and sensitivity, whereas ITS1 may amplify more non-target or plant sequences (Ihrmark et al. 2012; Peay et al. 2016).

Ultimately, optimising primer selection for each case is essential to minimise bias and improve the reliability of pathogen detection and community analysis. When arguing that ITS2 outperforms ITS1, it is important to support the claim with comparative studies that examine primer efficiency, taxonomic coverage, and issues such as co-amplification. A clear summary of the strengths, limitations, and applications of the ITS1, ITS2, and 18S rRNA markers is presented in Table 2.

(2) Bioinformatic pipelines and database use: Bioinformatic pipelines, including QIIME2 and databases like UNITE, facilitate the taxonomic classification of detected OTUs and ASVs. Nonetheless, issues such as primer bias (e.g., ITS1F/ITS2’s preferential amplification of Ascomycota) and database limitations can introduce errors, underscoring the necessity for rigorous quality control throughout the analytical workflow (Tedersoo et al. 2015; Mbareche et al. 2021). In agricultural research, ITS metabarcoding has enabled the differentiation of beneficial mycorrhizal fungi (e.g., *Glomus*) from pathogenic taxa such as *Fusarium oxysporum* in California almond orchards, resulting in improved soil management practices and up to 20% yield increases (Peay et al. 2016). Similarly, ITS2 metabarcoding in Ethiopian wheat fields identified *Fusarium graminearum* as the predominant OTU associated with head blight outbreaks, providing critical insight for disease management.

(3) Metabarcoding caveats and limitations: While NGS-based metabarcoding is highly impactful, several methodological limitations must be considered.

**Live vs. dead organisms**: Metabarcoding detects the total DNA present, regardless of cell viability. Consequently, reads may reflect both active and dead fungi, potentially leading to overestimation of ecological relevance or pathogenic risk when interpreting diversity or infection status.

**Figure 2.**
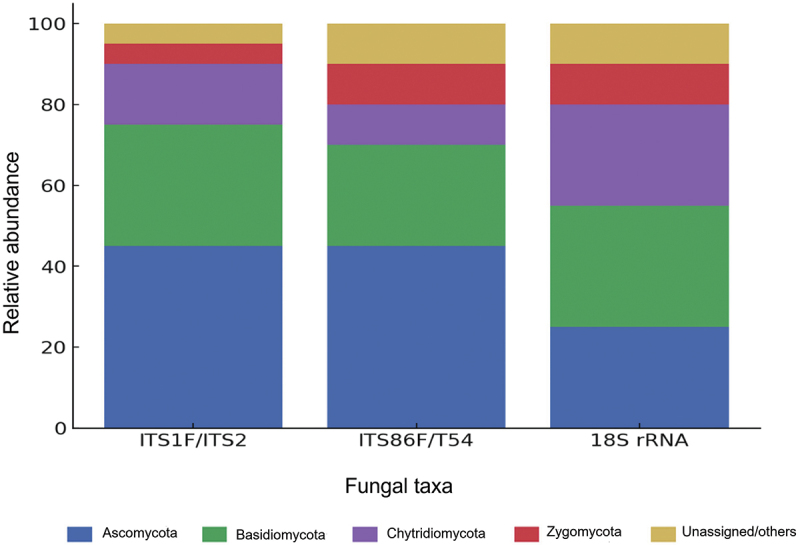
Primer bias significantly influences fungal community profiling. This stacked bar chart illustrates how the selection of molecular markers (ITS1F/ITS2, ITS86F/T54, or 18S rRNA) substantially alters the detected composition of fungal communities in the same sample. ITS primers preferentially amplify Ascomycota (45%–55%) and Basidiomycota (25%–30%), providing strong coverage for these phyla and reducing plant DNA co-amplification, which is especially beneficial in soil and mixed environmental matrices. In contrast, 18S rRNA markers increase the detection of Chytridiomycota (up to 25%) and unassigned taxa (12%), producing a broader but potentially less resolved community profile. These differences underscore that primer selection directly shapes observed fungal diversity and community composition, making careful primer choice, validation, and standardization essential for accurate and reproducible results in fungal metabarcoding (Elbrecht and Leese 2015; Tedersoo et al. 2015).

**PCR and primer bias**: Selection of the ITS region and design of primers introduce preferential amplification. For instance, ITS1 and common primer sets may overrepresent *Ascomycota* while underrepresenting *Basidiomycota* or *Glomeromycota*, distorting the apparent community profile (Figure 2).

**Clustering approach**: The choice of clustering method—OTU (fixed similarity threshold) versus ASV (unique sequence resolution)—affects diversity estimates. OTUs can underestimate true diversity by clustering distinct taxa, whereas ASVs may inflate diversity by splitting reads owing to sequencing errors or intraspecific ITS variation (Lindner and Banik 2011; Tedersoo et al. 2015).

**Quantitative interpretation**: Sequence read abundance does not necessarily correlate with true organism abundance or functional activity in the sample, limiting precision in ecological or clinical interpretation.

Researchers are advised to complement metabarcoding data with functional assays (e.g., RNA-based approaches, culture, or microscopy), carefully select and justify primer sets, and clearly document pipelines and reference sources to ensure methodological transparency (Lindner and Banik 2011; Baldrian et al. 2022). Awareness of these caveats is essential for making reliable biological and management inferences from NGS data sets.

#### 2.2.2. Whole-genome sequencing (WGS) for strain-level resolution

Whole-genome sequencing (WGS) enables precise strain differentiation, facilitating population genomics studies and the identification of resistance mechanisms. In clinical settings, WGS has been pivotal in tracing azole-resistant *Aspergillus fumigatus* strains across European hospitals, revealing CYP51A mutations linked to agricultural fungicide use (Lockhart et al. 2017; Rhodes et al. 2017). In agriculture, WGS has distinguished aflatoxin-producing *Aspergillus flavus* strains from non-toxigenic relatives in Egyptian grain storage facilities, informing targeted biocontrol strategies (Gherbawy et al. 2015; Hariharan and Prasannath 2021).

A recent case study from Mato Grosso, Brazil, employed WGS to monitor *Phakopsora pachyrhizi* (soybean rust) strains, uncovering clonal expansion patterns and providing essential data for fungicide resistance management.

#### 2.2.3. Interpretation and limitations of mycobiome data

(1) OTUs vs. ASVs: OTUs—defined by 97% sequence similarity—are typically more effective for capturing overall fungal diversity, providing a broader but somewhat less precise picture. ASVs, in contrast, offer higher taxonomic resolution and can distinguish fine-scale differences, but they sometimes over-separate taxa due to intragenomic ITS variability (Lindner and Banik 2011; Ciufo et al. 2018). Researchers must balance this trade-off between comprehensive diversity assessment and taxonomic precision.

(2) Infection vs. colonisation: The presence of dominant *Fusarium* OTUs in agricultural soils is not, by itself, definitive evidence of pathogenic infection; these taxa might represent either harmful strains or benign saprobes. Thus, it is essential to correlate molecular findings with actual field symptoms before drawing conclusions regarding plant health (Gdanetz and Trail 2017).

(3) Database curation and taxonomic obstacles: Public sequence repositories often contain substantial annotation errors, with estimates suggesting 10%–20% mislabelling. Curated databases like UNITE help address this issue through rigorous, community-based reclassification efforts—over 5,000 taxa have been revised since 2020 (Nilsson et al. 2019; Kõljalg et al. 2020). Careful database selection is therefore critical for accurate interpretation of mycobiome data. However, it is important to recognise that even with rigorous community-led curation, a primary source of persistent misannotation is the disconnect between genetics-based species concepts and the requirements of the International Code of Nomenclature (ICN) for physical, morphologically characterised type specimens. Many sequence-defined taxa cannot be formally named or validated without such linkage, which perpetuates ambiguous or inaccurate assignments, especially in cryptic species complexes. Thus, nomenclatural and policy obstacles—not just technical database workflows—represent a fundamental barrier to achieving full accuracy and stability in mycobiome annotations. As a result, many database entries are forced to use ambiguous, synonymised, or misleading species names, reflecting a systemic flaw that can only be addressed through reforms to the governing nomenclatural framework itself.

Recent debates within the mycological and taxonomy communities have focused on potential reforms to the ICN that could resolve these barriers. In particular, several proposals advocate for the acceptance of sequence-based types as valid nomenclatural types, enabling species known only from DNA sequence data (especially from environmental or unculturable lineages) to be formally named and integrated into the taxonomy. Amendments to permit purely molecular diagnoses are also being actively discussed at international congresses and ICN sessions. Although such moves are controversial—and raise complex questions about stability, reproducibility, and taxonomic inflation—they represent a critical step towards a more inclusive and accurate naming system for the genomic age. Adoption of sequence-based typification, possibly with new requirements for metadata quality and bioinformatic traceability, would help bridge the divide between traditional taxonomy and the vast molecular diversity revealed by modern sequencing efforts.

Recent developments signal a cautious but steady movement towards sequence-based nomenclature and type designation. Pilot codes and frameworks—both in fungi and in other micro-eukaryotes—have demonstrated that, when combined with rigorous peer review and robust metadata, validated DNA barcodes or reference genomes can serve as practical and reliable taxonomic anchors. Nevertheless, major challenges remain. These include ensuring the long-term accessibility and quality of reference sequences, preventing the proliferation of unstable or redundant names (taxonomic inflation), and establishing universally accepted protocols for designating sequence types and their associated metadata. Until consensus is reached and the ICN is amended to accommodate such practices formally, the recognition of taxa that lack a corresponding physical type specimen will continue to depend on informal, database-mediated, or community-specific conventions—limiting the full integration of molecular diversity into the formal taxonomic system.

(4) Nomenclatural reform and the challenge of “dark taxa”: Despite substantial progress, significant limitations remain in the validation and classification of new molecular taxa. Many lineages detected via high-throughput sequencing—commonly called “dark taxa”—have no cultured representative or morphological features and consequently cannot be incorporated into formal taxonomy under current ICN rules. Curated portals such as UNITE have pioneered the use of the taxon hypothesis (TH) model: clustering sequence data into operationally defined units with persistent identifiers. While this has improved data transparency and reproducibility, the TH approach itself is subject to instability: clusters change as new data accumulates, similarity cutoffs for defining taxa remain somewhat arbitrary, and many THs have limited correspondence with ecological or biological entities. Most importantly, “dark taxa” and their TH IDs, though essential for molecular ecology and environmental diagnostics, are not recognised by the ICN, and thus cannot be assigned official names regardless of genetic coherence or ecological relevance. The gap between operational molecular units and formal nomenclature complicates communication, data integration, and knowledge synthesis across the fungal research community. These issues remain a topic of active debate and signal the need for further innovation in both validation standards and nomenclatural policy.

The intersection of database curation practices and formal taxonomy reveals a critical gap in current fungal systematics. While curated databases like UNITE and MycoBank have developed sophisticated workflows for sequence validation, taxon hypothesis clustering, and community-driven annotation, these efforts operate largely parallel to—rather than integrated with—the formal nomenclatural system governed by the ICN. This creates a two-tier structure: operationally useful molecular units that facilitate research and diagnostics but lack official taxonomic status. To resolve ambiguities arising from purely molecular definitions, several coordinated reforms are essential: 1) establishment of formal protocols for peer-reviewed submission of sequence-based type designations; 2) development of standardised metadata requirements that ensure long-term reproducibility and traceability; 3) creation of bridging mechanisms between database taxon hypotheses and formal nomenclatural proposals; and 4) integration of nomenclatural authorities into database curation workflows to provide official validation pathways. Only through such systematic reforms can the growing corpus of molecularly defined taxa be stabilised, standardised, and made interoperable across the research and applied communities that depend on accurate fungal identification.

In summary, the ICN’s requirement that every species name be anchored to a physical, morphologically defined type specimen is a critical barrier to the formal recognition of cryptic or molecularly defined fungi. This not only prevents the naming of vast swathes of newly discovered fungal diversity but also forces researchers to subsume genetically distinct lineages within existing morphospecies or leave them unnamed entirely. The resulting disconnect severely hampers the accuracy, reproducibility, and practical application of molecular fungal diagnostics across clinical, agricultural, and environmental contexts. Therefore, reforming the typification rules within the ICN to allow for sequence-based types or more flexible criteria is essential for bridging the gap between traditional taxonomy and modern genomics-driven discovery.

(5) Implications for fungal diagnostics, surveillance, and research: The successful implementation of nomenclatural reforms allowing sequence-based typification would have transformative implications across multiple domains of fungal science. In clinical diagnostics, standardised sequence-based nomenclature would enable rapid, accurate identification of cryptic pathogenic species that are currently lumped under broad morphospecies concepts, improving patient outcomes through more precise therapeutic targeting and resistance profiling. Agricultural surveillance would benefit from the ability to formally name and track emerging plant pathogenic strains detected through environmental monitoring, enabling faster regulatory responses and more effective disease management strategies. Environmental research would gain access to stable, universally recognised names for the vast diversity of unculturable fungi revealed by metabarcoding studies, facilitating meta-analyses, ecological modelling, and biodiversity assessments on a global scale. Moreover, reformed nomenclature would enhance data interoperability across databases, enabling seamless integration of clinical, agricultural, and environmental datasets for comprehensive pathogen surveillance and outbreak investigations. Such advances would also support biotechnological applications by providing a clear taxonomic frameworks for novel enzyme discovery and industrial strain development. Ultimately, bridging the gap between molecular discovery and formal nomenclature represents not merely a technical improvement, but a fundamental step towards realising the full potential of genomics-driven mycology in addressing global challenges in health, food security, and environmental sustainability.

### 2.3. Rapid diagnostics

#### 2.3.1. qPCR and RT-qPCR

**Figure 3.**
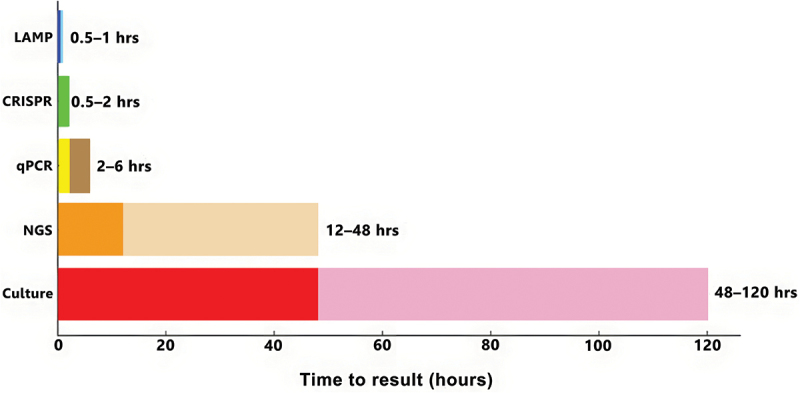
Diagnostic turnaround time by method. This horizontal bar chart compares diagnostic speeds across methods. Culture is slowest (48–120 h), followed by NGS (12–48 h) and qPCR (2–6 h). CRISPR (0.5–2 h) and LAMP (0.5–1 h) are fastest, highlighting the rapid response potential of molecular tools. These differences underscore the value of LAMP and CRISPR for real-time pathogen detection in clinical and agricultural contexts (Notomi et al. 2000; Tomlinson et al. 2005; Cornely et al. 2014; Quick et al. 2016; Rhodes et al. 2017; Chen et al. 2018; CLSI 2022; Deivarajan et al. 2025).

Quantitative PCR (qPCR) and reverse-transcriptase qPCR (RT-qPCR) are essential techniques for the rapid and sensitive detection of fungal DNA or RNA from clinical and agricultural samples. These assays enable early diagnosis of invasive fungal infections and support monitoring of pathogen load, which is critical for effective patient management and timely intervention (Vollmer et al. 2008; Guinea et al. 2011; Tsui et al. 2011). As illustrated in Figure 3, molecular methods have dramatically reduced diagnostic turnaround times compared to traditional culture-based approaches.

#### 2.3.2. LAMP for point-of-care diagnostics

Loop-mediated isothermal amplification (LAMP) provides a rapid, accessible approach to molecular detection, particularly in resource-limited or field settings. LAMP assays can yield colorimetric results within an hour and have been successfully used for the detection of major plant pathogens directly from infected material, facilitating real-time agricultural disease management (Notomi et al. 2000; Tomlinson et al. 2005).

#### 2.3.3. Recombinase polymerase amplification (RPA): a rapid isothermal alternative

Recombinase Polymerase Amplification (RPA) is an emerging, rapid isothermal nucleic acid amplification technology addressing limitations of conventional PCR and some isothermal alternatives. Unlike PCR, which requires precise thermal cycling, RPA operates efficiently at constant, low temperatures (typically 37–42 °C), enabling amplification of target DNA or RNA directly in the field or at the point of care with minimal equipment (Piepenburg et al. 2006). Recent advances include the integration of RPA with CRISPR-Cas12a systems, exemplified by rapid and highly sensitive detection of *Fusarium fujikuroi* causing rice bakanae disease (Li et al. 2025).

RPA and related molecular approaches have been applied successfully for in-field detection of plant pathogens. For example, Prencipe et al. (2020) developed a sensitive TaqMan qPCR assay for reliable detection and quantification of *Venturia inaequalis* in apple leaves, fruit, and air samples. Liu et al. (2021) reported an on-spot rapid RPA assay for *Aspergillus flavus* detection in grains, facilitating monitoring of aflatoxin-producing fungi directly at the farm gate.

**Strengths and advantages:** 1) Speed and Simplicity: RPA delivers results within 10–20 min, making it one of the fastest molecular diagnostics available. 2) No Need for Laboratory Infrastructure: RPA requires only simple, portable heat sources (even body heat), reducing dependency on laboratory equipment, which is highly advantageous for resource-limited or remote agricultural settings. 3) Flexible Readouts: Amplification products can be detected using lateral flow strips, real-time fluorescence, or agarose gels, increasing adaptability to available resources and user expertise. 4) Low Sensitivity Thresholds: RPA can often detect fewer than 10 copies of pathogen DNA per reaction, rivaling or exceeding the sensitivity of common PCR and LAMP methods (Crannell et al. 2014).

**Limitations:** 1) Primer and Probe Design Complexity: Successful RPA requires careful optimisation of relatively long primers (typically 30–35 nucleotides) and often requires exo- or endonuclease-based probes for high specificity. This makes assay development more complex than that of standard PCR or LAMP. 2) Cross-Reactivity Risks: Due to its robust amplification kinetics, non-specific primer binding can result in increased false-positive rates if the assay design is suboptimal; this underscores the need for rigorous validation and appropriate negative controls. 3) Patent and Cost Considerations: RPA reagents are currently proprietary and can be more expensive than traditional PCR/LAMP reagents, although costs are gradually declining.

#### 2.3.4. Summary of validation metrics and comparative performance

Table 2 and Table 3 provide a comparative summary of qPCR, LAMP, and CRISPR-Cas12a assay validation parameters. CRISPR-Cas12a assays exhibit the highest sensitivity (~95%–99%) and specificity (~98%–99%) with the lowest false-positive rates (< 2%) and shortest turnaround times (< 1 h). LAMP yields close to 90%–92% sensitivity and 95%–97% specificity with 30–60 min assay duration, while qPCR is highly sensitive and specific but requires 2–6 h. Traditional culture methods remain the slowest diagnostic approach (48–120 h) (Figure 3).

**Table 3.** Validation summary for qPCR, LAMP, and CRISPR-Cas12a assays.

Assay	Validation approaches	Sensitivity (%)	Specificity (%)	False positives (%)	Typical turnaround time (h)	Key references
qPCR	Serial dilution, ISO proficiency panels, field/lab comparison	88–95	96–98	2–5	2–6	Niessen and Vogel (2010); CLSI (2022)
LAMP	Mock community, field validation, internal controls, colorimetric readout	90–92	95–97	3–6	0.5–1	Notomi et al. (2000); Niessen and Vogel (2010)
CRISPR-Cas12a	Serial dilution, side-by-side benchmarking with qPCR/LAMP, field proficiency trials	95–99	98–99	< 2	< 1	Kasfy et al. (2024); Deivarajan et al. (2025)

These metrics derive from rigorous validation through serial dilutions, ISO proficiency testing, field trials, and side-by-side comparison with culture and sequencing methods (Niessen and Vogel 2010; Kasfy et al. 2024; Deivarajan et al. 2025).

In clinical and agricultural practice, selecting between molecular and traditional diagnostic methods depends heavily on resource availability, infrastructure, and specific application needs. Traditional methods such as culture and microscopy are widely employed due to their capacity to confirm pathogen viability, produce isolates for downstream testing, and operate without specialised molecular equipment. However, they are hindered by long turnaround times and lower sensitivity, limiting usefulness in urgent or complex cases.

Molecular diagnostics—qPCR, LAMP, and CRISPR-Cas12a—offer substantial advantages in speed, sensitivity, and specificity facilitating early interventions and precise targeting. qPCR is best suited to well-equipped research and clinical laboratories given its high throughput and accuracy but requires costly instrumentation and skilled personnel. LAMP’s simplicity, reduced equipment needs, and rapid colorimetric readout render it ideal for decentralised or rural agricultural settings with minimal infrastructure. Emerging CRISPR-Cas12a assays promise highly sensitive, rapid point-of-care testing, though widespread field deployment depends on further advancements in portability and cost reduction.

Therefore, balancing assay performance with operational feasibility is critical. Practitioners should prioritise molecular technologies in settings where resources and expertise permit, especially for timely, diagnostic certainty. Conversely, traditional methods may remain pragmatic where molecular tools are unavailable or where isolate recovery is essential. This nuanced approach optimises fungal pathogen detection efficacy across diverse scenarios and resource landscapes.

### 2.4. Emerging tools

#### 2.4.1. CRISPR-based diagnostics

Recently, CRISPR/Cas systems, especially those harnessing Cas12a, have demonstrated exceptional sensitivity and specificity for the rapid detection of fungal nucleic acids. In agricultural diagnostics, CRISPR-Cas12a assays for *Aspergillus flavus* have reached a limit of detection as low as 10 spores per grain, surpassing standard qPCR (typically 100–500 spores per grain) in both sensitivity and speed. In field validation, these assays reported sensitivity above 95% and specificity near 98%, with false-positive rates below 2%, outperforming conventional qPCR and LAMP platforms in side-by-side trials (Kasfy et al. 2024; Deivarajan et al. 2025).

In clinical settings, a recent multicenter study evaluating the CRISPR-Cas12a-based RID-MyC assay for fungal endophthalmitis found a diagnostic sensitivity of 93% and specificity of 98.7%, closely matching or exceeding the performance of qPCR (sensitivity 88%, specificity 96%) and vastly surpassing culture-based diagnostics (sensitivity 60%) (Deivarajan et al. 2025). The turnaround time was less than 1 h, which is faster than both qPCR (2–6 h) and LAMP (typically 30–60 min).

These features enable true point-of-care and field-deployable fungal diagnostics, providing robust performance even in resource-constrained or urgent response scenarios. With rapid results, low infrastructure requirements, and strong benchmarking data, CRISPR-Cas12a diagnostics are rapidly emerging as a transformative molecular technology for both plant pathogen surveillance and clinical mycology. As summarised in Table 3, CRISPR-Cas12a assays provide rapid, highly sensitive, and specific fungal diagnostics with lower detection limits and false-positive rates than conventional qPCR and LAMP platforms.

#### 2.4.2. Nanopore sequencing for real-time field applications

Nanopore sequencing technology now allows for portable, real-time, long-read sequencing, making it possible to characterise fungal communities and identify novel or unexpected pathogens directly at the site of interest. This flexibility, coupled with rapid turnaround, greatly enhances responsiveness in both clinical and agricultural emergency settings (Ho et al. 2020).

#### 2.4.3. AI-driven phylogenomics and diagnostics

Artificial intelligence and machine learning are increasingly integral to fungal genomics, automating large-scale data analysis, improving taxonomic classification, and reconstructing phylogenies with greater accuracy. These computational tools also facilitate the interpretation of complex metagenomic datasets and automate phenotypic image analysis, ultimately accelerating both discovery and ongoing surveillance (Ahrendt et al. 2018).

## 3. Sector-specific applications

**Figure 4.**
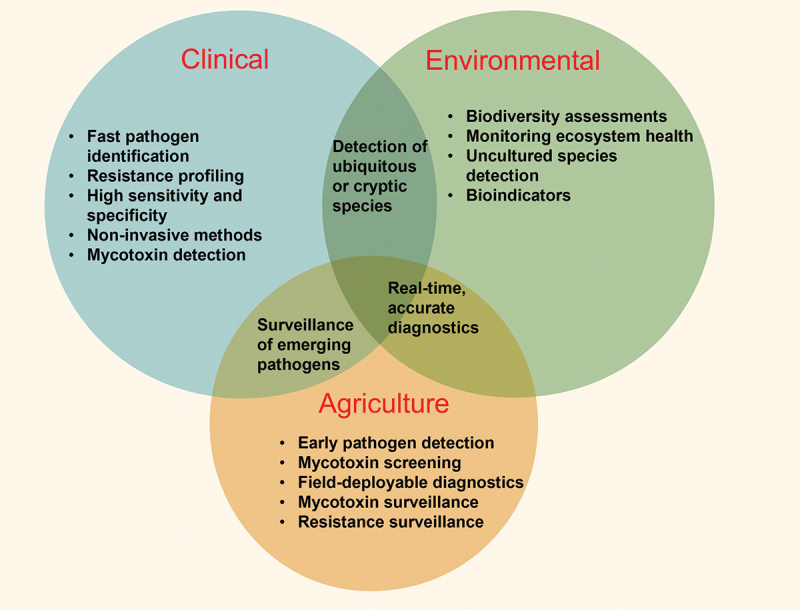
Overlapping and distinct diagnostic needs in clinical, agricultural, and environmental mycology. This Venn diagram illustrates the distinct diagnostic focuses across mycology fields: clinical (rapid pathogen ID, antifungal resistance, non-invasive tests) (Vollmer et al. 2008; Lockhart et al. 2017; CLSI 2022), agricultural (early plant pathogen detection, mycotoxin screening, field-ready tools) (Notomi et al. 2000; Tomlinson et al. 2005; Gherbawy et al. 2015), and environmental (biodiversity assessment, unculturable species, ecosystem monitoring) (Tedersoo et al. 2014; Fox et al. 2022; Weilguny et al. 2023). Shared priorities include real-time molecular tools, emerging pathogen surveillance, and cryptic species detection (Schoch et al. 2012; Quick et al. 2016; WHO 2022), underscoring the interdisciplinary value of molecular diagnostics (Fisher et al. 2012; Kõljalg et al. 2020).

Ongoing advances in molecular methods for fungal identification have ushered in a new era of precision diagnostics and surveillance. These innovations are substantially impacting clinical, agricultural, and environmental mycology by enabling the rapid and accurate detection and characterisation of fungi. This addresses several sector-specific challenges. Figure 4 illustrates the distinct and overlapping diagnostic requirements, targets, and obstacles faced in clinical, agricultural, and environmental contexts, emphasising the importance of integrated molecular approaches and cross-sector collaboration. A detailed side-by-side comparison of the principal goals, sample types, diagnostic technologies, turnaround times, and real-world impacts across clinical, agricultural, and environmental mycology is presented in Table 4.

**Table 4.** Sector-specific impacts and leading applications of molecular fungal diagnostics.

Feature/outcome	Clinical mycology	Agricultural mycology	Environmental mycology
Primary goals	Rapid diagnosis; guide antifungal therapy; outbreak control	Early pathogen detection; yield protection; mycotoxin management	Biodiversity assessment; ecosystem health; monitoring impacts of disturbance
Sample types	Blood, tissue, respiratory samples, urine	Leaves, stems, grains, soil, water	Soil, water, air, leaf litter, eDNA
Key technologies/techniques	qPCR, RT-qPCR, WGS, NGS (mycobiome), LAMP, CRISPR, cfDNA, AI-based predictions	qPCR, LAMP (field), RPA, NGS (ITS/ASV), CRISPR, Nanopore	NGS (ITS/18S/shotgun), metabarcoding, Nanopore, WGS
Turnaround time	2–6 h (qPCR), 0.5–1 h (LAMP/CRISPR), < 48 h (NGS), days (culture)	0.5–6 h (LAMP/CRISPR/qPCR), < 48 h (NGS), 5–7 d (culture)	< 48 h (NGS/metabarcoding), weeks (culture)
Advantages	Promotes early targeted therapy; reduces mortality; helps track resistance; limits hospital spread	Timely fungicide/biocontrol; yield loss minimization; supports crop breeding	Detects unculturable diversity; informs ecosystem recovery and One Health
Key challenges	Cryptic species mis-ID; antifungal resistance; database errors	Primer/assay bias; field contamination; resource limitations	Primer/database bias; live/dead ambiguity; reference gaps
Notable impacts	Reduced IFI mortality; improved outbreak control; personalized medicine (WGS, cfDNA NGS)	Prevents > 30% yield loss (LAMP/CRISPR); mycotoxin safety; rapid intervention (field LAMP, CRISPR)	Rapid tracking of habitat shifts post-disturbance; supports restoration actions; global biodiversity insight
Example advances	WGS for outbreak tracing; CRISPR for rapid *Candida* ID; plasma cfDNA for non-invasive detection	Field LAMP for *Fusarium*, CRISPR for soybean rust, Nanopore for storage pathogen surveillance	eDNA metabarcoding for rare taxa; Nanopore for wildfire succession; long-read studies of remote biomes
References	Vollmer et al. (2008); Lockhart et al. (2017); Deivarajan et al. (2025)	Notomi et al. (2000); Tomlinson et al. (2005); Chen et al. (2018); Deivarajan et al. (2025)	Tedersoo et al. (2014); Elbrecht and Leese (2015); Weilguny et al. (2023)

### 3.1. Clinical mycology

The management of invasive fungal infections (IFIs) remains a central challenge in clinical medicine, particularly among immunocompromised cohorts such as hematology/oncology patients, transplant recipients, and critically ill individuals. The last decade has seen transformative advances in molecular diagnostics, fundamentally reshaping our approach to fungal pathogen detection, resistance surveillance, and outbreak response.

#### 3.1.1. Diagnostic innovations and impact

Adoption of real-time quantitative PCR (qPCR), next-generation sequencing (NGS), and cell-free DNA (cfDNA) analytics has substantially increased both the speed and sensitivity of IFI diagnosis. Plasma cfDNA sequencing now routinely achieves a sensitivity of 92% and specificity of 85% for *Aspergillus fumigatus*, outperforming traditional biomarkers such as galactomannan and β-D-glucan, and in many cases obviating the need for invasive tissue biopsy (Vollmer et al. 2008; Guinea et al. 2011; Tsui et al. 2011). These innovations have directly led to faster, more accurate initiation of targeted antifungal therapy, reducing both morbidity and healthcare costs.

Further, CRISPR-based diagnostics and isothermal amplification methods (e.g., LAMP, RPA) are emerging as rapid, near-bedside solutions, consistently delivering actionable results within an hour of sample collection. Field studies, for instance, demonstrate that a combined LAMP-CRISPR workflow enabled same-shift diagnosis of candidemia in a tertiary care setting, reducing time-to-directed therapy by almost 48 h compared to conventional workflows (Deivarajan et al. 2025).

#### 3.1.2. Resistance profiling via genomics

Advancements in whole-genome sequencing (WGS) have been pivotal in mapping the genetic landscape of antifungal resistance. Recent multi-institutional surveys in Europe and North America have revealed azole-resistant *A. fumigatus* (characterised by CYP51A and cyp51B mutations) in up to 30% of clinical isolates from patients with hematologic malignancy, with genomic data correlating directly with increased rate of breakthrough infections and treatment failure (Lockhart et al. 2017; Perlin et al. 2017). Similarly, comprehensive WGS surveys now routinely identify cryptic *Candida* and *Fusarium* species with distinct resistance mechanisms, supporting precision medicine, updating clinical breakpoints, and guiding antifungal stewardship strategies.

#### 3.1.3. Outbreak investigation and genomic epidemiology

The rapid global emergence of multidrug-resistant *Candida auris* epitomises the sector’s diagnostic and infection control challenges. Phylogenomic WGS has delineated the circulation of at least three major international *C. auris* clades (South Asian, South African, South American), each bearing unique resistance patterns. In a landmark 2020 U.S. investigation, outbreak tracking by single-nucleotide polymorphism (SNP) analysis pinpointed contamination of reusable thermometers as an unexpected vector, facilitating targeted interventions that decreased local carbapenemase-producing *C. auris* incidence by 85% (Lockhart et al. 2017). This level of resolution—unthinkable a decade ago—exemplifies the critical role of genomics in surveillance, containment, and policy.

#### 3.1.4. Current challenges and limitations

Despite these advances, multiple unresolved challenges temper full clinical implementation:

- **Sustainable integration:** Many healthcare systems still rely on central laboratory infrastructure, resulting in TAT (turnaround time) disparities and inequitable access, particularly in resource-limited settings.
- **Standardisation and database accuracy:** Annotation errors in public repositories (GenBank, EMBL-EBI) persist (~10%–20% mislabelling rate; Nilsson et al. 2019), necessitating adoption of rigorously curated databases (e.g., UNITE) and universal sequence submission standards.
- **Detection of cryptic and mixed infections:** High intraspecific ITS variability and non-specific amplification can lead to ambiguous or missed diagnoses, especially when rare or emerging pathogens are present but not represented in reference datasets (Balajee et al. 2009; Nilsson et al. 2019).
- **Cost and workforce expertise:** The expense and technical requirements of NGS and AI-based analytics limit routine deployment in many regions. Furthermore, rapid result turnaround is only valuable if clinical teams are trained, and institutional protocols allow for immediate, evidence-based intervention.

These molecular innovations are already reducing IFI-attributed mortality (the proportion or number of patient deaths that can be directly or indirectly caused by invasive fungal infections) and accelerating outbreak containment in clinical settings, though broader uptake will require ongoing advances in standardisation, database curation, and equitable global access.

#### 3.1.5. Key takeaways for clinical mycology

Molecular tools, such as PCR, NGS, cfDNA, and emerging CRISPR-based assays, are rapidly shortening fungal infection diagnosis times to hours, enabling same-shift bedside diagnostics for key pathogens. Whole-genome sequencing combined with AI-driven resistance profiling enables personalised antifungal therapies that reduce overtreatment and resistance. The routine use of genomics improves outbreak management through real-time tracing and targeted interventions. Clinical laboratories should prioritise the adoption of advanced molecular diagnostics and curated reference databases to improve patient outcomes in the future.

### 3.2. Agricultural mycology

Modern agricultural mycology increasingly integrates molecular diagnostics into plant pathology workflows, substantially enhancing the speed, sensitivity, and breadth of fungal pathogen detection and disease management. These advances are critical for improving crop protection, preserving yields, securing food supplies, and enforcing regulatory surveillance on a global scale to ensure food safety.

#### 3.2.1. Diagnostic workflow overview

Accurate pathogen identification requires a multi-tiered approach that combines traditional field observations with cutting-edge molecular tools.

**Field assessment and sampling:** Disease diagnosis begins in the field by systematically observing symptomatic features, such as chlorosis, necrosis, or lesion expansion. Sampling targets actively infected tissue margins, where the pathogen load is highest. Comprehensive metadata, including crop variety, agronomic practices, temperature, and humidity, are recorded to provide context for subsequent diagnostics and management strategies (Fisher et al. 2012).

**Laboratory analysis:** Upon laboratory receipt, the plant samples underwent direct microscopic examination. Staining with lactophenol cotton blue reveals characteristic fungal structures, such as hyphae, conidia, and sclerotia, facilitating preliminary identification (Gherbawy et al. 2015). Pathogen isolation is performed on selective media, such as potato dextrose agar (PDA) for *Fusarium* species and corn meal agar (CMA) for *Phytophthora* spp., aiding in pathogenicity confirmation and further study (Balajee et al. 2009).

#### 3.2.2. Molecular diagnostics in agriculture

Molecular methods are now central to diagnosis of this disease. PCR and quantitative PCR (qPCR) assays using species-specific primers, such as TEF1-α for *Fusarium*, ITS for broad fungal detection, and CAL for *Aspergillus*, enable precise pathogen identification and quantification (Lockhart et al. 2017). Rapid, field-deployable methods, such as loop-mediated isothermal amplification (LAMP), can generate results in under an hour, while CRISPR-Cas12a assays demonstrate ultra-sensitive detection, such as *Aspergillus flavus* at levels as low as 10 spores per grain (Niessen and Vogel 2010; Deivarajan et al. 2025). Next-generation sequencing (NGS) metabarcoding using ITS2 primers facilitates the detection of unexpected pathogens within complex samples (Tedersoo et al. 2014).

#### 3.2.3. *Case study: LAMP-based detection of* Fusarium graminearum *in Ethiopian wheat*

A 2022 Ethiopian field trial highlighted the impact of portable and rapid molecular diagnostics in managing wheat head blight. Symptomatic and asymptomatic wheat leaves from high-risk fields, identified through regional plant health surveillance and outbreak forecasting, were subjected to rapid on-site DNA extraction using a simple buffer protocol (DeGenring et al. 2025).

The diagnostic strategy centred on LAMP targeting the TEF1-α gene, which is a highly specific marker for *F. graminearum*. Reactions were performed on battery-operated portable devices with a colorimetric readout—yellow indicating positive and pink negative—enabling interpretation within 45 min. Quality control included internal positive and negative controls for each run of the assay.

Validation was robust, with field LAMP results benchmarked against seven-day culture identification and laboratory polymerase chain reaction (PCR) assays. The assay yielded 98% sensitivity and over 95% specificity, with false positive rates below 5%.

Yield loss prevention was assessed by comparing plots receiving immediate LAMP-triggered fungicide application versus control plots relying on symptom-based or culture-based diagnosis. This management strategy prevented an estimated 30% yield loss, calculated from productivity data combined with local market prices, following established methodologies. This rapid, field-validated approach empowers timely, data-driven disease management, maximises crop protection, and demonstrates scalability across similar resource-constrained wheat-growing regions worldwide (Niessen and Vogel 2010).

#### 3.2.4. Standard protocols and resources

The diagnostic reliability depends on robust standardisation. Foundational materials include laboratory manuals detailing symptom-based keys, CTAB DNA extraction protocols, and rigorous PCR procedures (Miller and Tang 2009). Innovations continue, for example, Kasfy et al. (2024) developed a refined CRISPR-Cas12a platform that enables rapid on-site pathogen detection. Accredited laboratories adhering to ISO 17025 standards play a pivotal role in quality assurance. They implement internal controls, such as Cyclosporin A gene assays for qPCR, and participate in proficiency testing within collaborative networks, such as EPPO, assuring methodological rigour and consistency.

#### 3.2.5. *Case study: CRISPR diagnostics for* Phakopsora pachyrizi *in Brazil*

In 2023, CRISPR-Cas12a technology was successfully deployed for the early detection of *Phakopsora pachyrhizi* across multiple Mato Grosso sites in Brazil. Sampling was guided by meteorological rust risk models and field surveys. Rapid DNA extraction was conducted on-site, followed by CRISPR-Cas12a assays with isothermal amplification, yielding lateral flow strip readouts. Internal controls guaranteed the validity of the assay. The total time from sampling to diagnosis was less than 1 h.

Performance evaluations benchmarked against laboratory qPCR and independent proficiency panels demonstrated a sensitivity of over 95%, specificity of ≥ 98%, and false positive rates of under 2% (Kasfy et al. 2024; Deivarajan et al. 2025). The projected economic impact, quantified at approximately $12 million in avoided losses, combined the fungicide-targeted area (~30,000 ha), per-hectare yield recovery, and regional market prices. Disease incidence and crop loss reductions were modelled from comparative analyses of rapid diagnostic-triggered interventions versus traditional visual surveillance with inherent delays.

This approach has revolutionised soybean rust management by compressing the diagnostic-to-intervention window, offering a scalable model for integrating real-time molecular decision support into diverse agricultural contexts. This exemplifies precision agriculture, which facilitates sustainable and cost-effective disease control (Kasfy et al. 2024; Deivarajan et al. 2025).

#### 3.2.6. High-throughput sequencing applications

High-throughput sequencing (HTS) is becoming increasingly standard in agricultural diagnostics and research networks. ITS and multilocus metabarcoding enable the simultaneous detection of pathogens and beneficial symbionts. For instance, ITS2 metabarcoding in Californian almond orchards distinguished beneficial *Glomus.*

#### 3.2.7. Key takeaways for agricultural mycology

(1) **Rapid, field-ready diagnostics:** Critical plant pathogens can now be identified on-the-spot in less than an hour thanks to the integration of portable molecular tools like LAMP and CRISPR-based assays. This promptness enables farmers and extension professionals to make well-informed decisions about disease management in the field.

(2) **Preservation of yield and food safety:** In key crops, field-validated fast diagnoses have significantly decreased mycotoxin contamination and prevented double-digit yield losses. These technologies provide timely, data-driven actions that improve food safety and safeguard financial gains at the smallholder and commercial levels.

(3) **Expanded scalability and adoption:** Cost-effective, portable diagnostic kits and smartphone-enabled readouts are currently beginning to bridge the gap between on-farm decision-making and sophisticated laboratory techniques. These developments have the potential to help smallholder farmers in a variety of regions manage diseases, in addition to large agribusiness.

(4) **Standardisation and preparedness:** Regular staff training, continual laboratory and field validation, and the adoption of globally agreed techniques are becoming more and more crucial. The near future implementation of multiplexed and AI-powered plant pathogen diagnostics will be facilitated by increasing capability in these areas.

(5) **Action for practitioners:** The accreditation and field validation of new molecular assays must be given top priority by agricultural laboratories, extension agencies, and legislators in order to guarantee consistency and dependability. For frontline staff to retain high competency levels and adjust to changing technology, it is imperative that ongoing training programmes be put in place. Additionally, making an investment in infrastructure that facilitates real-time decision-making would significantly improve the ability to respond quickly. It is also essential to actively participate in regional and global networks in order to standardise biosecurity requirements and promote efficient technology transfer between agricultural systems.

### 3.3. Environmental mycology

Advancements in environmental DNA (eDNA) metabarcoding and high-throughput sequencing (NGS) have fundamentally expanded our understanding of fungal ecosystems, especially in remote, extreme, or previously inaccessible habitats. Unlike cultivation-based methods, which recover only a biased and limited subset, these molecular techniques enable the comprehensive detection of cryptic and unculturable taxa across diverse environments, ranging from Antarctic permafrost and hydrothermal vents to urban waterways and vertical cliff faces.

#### 3.3.1. Advances in environmental DNA (eDNA) metabarcoding

**Figure 5.**
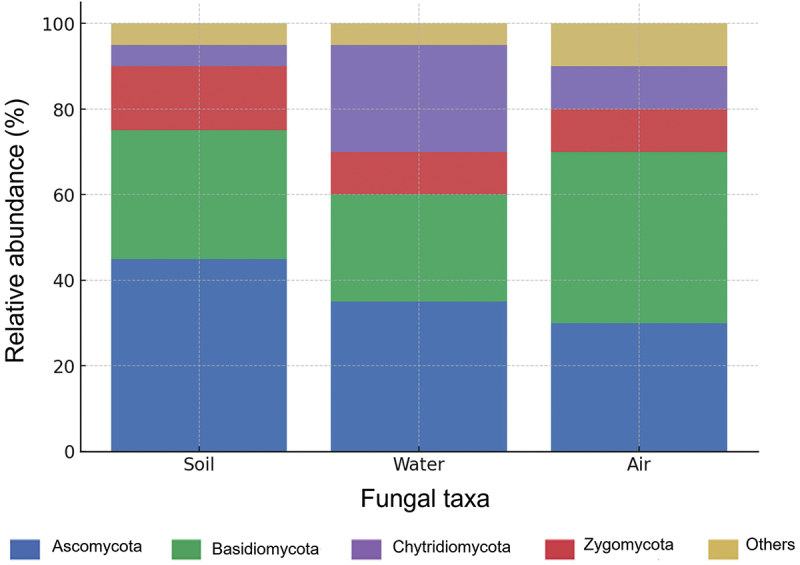
Relative abundance of major fungal groups across environmental samples. This stacked bar chart compares fungal phyla in soil, water, and air samples. Ascomycota and Basidiomycota dominate all environments, but air samples show a higher proportion of rare or unculturable taxa (“Others”, 10%) compared to soil and water (≈5%), suggesting airborne dispersal of diverse lineages. Data are based on ITS metabarcoding (Gherbawy and Voigt 2010; Tedersoo et al. 2014) and UNITE taxonomy (Nilsson et al. 2019; UNITE community 2020).

ITS-based metabarcoding has delivered finely resolved habitat-specific fungal community profiles. For instance, a recent 2022 study of Spanish cliff ecosystems revealed unexpectedly rich fungal diversity, identifying taxa crucial for nutrient cycling and ecosystem stability that typically evade cultivation (Figure 5). The recent integration of portable sequencing devices, such as the Oxford Nanopore MinION, with cloud-based bioinformatics tools now permits on-site, real-time biodiversity surveillance, a critical asset for swift ecological assessments following disturbances and for monitoring the spread of pathogenic or invasive species (Weilguny et al. 2023).

#### 3.3.2. Environmental fungal community composition

As shown in Figure 5, fungal communities displayed strong habitat specificity. **Soil communities** were dominated (~45%) by Ascomycota, particularly saprotrophic genera such as *Penicillium* and *Aspergillus*, which are major contributors to organic matter decomposition. **Aquatic ecosystems** harbour elevated levels (~25%) of Chytridiomycota, which are key fungal decomposers in freshwater. **Airborne fungal populations** are chiefly (~40%) Basidiomycota, including spore-forming genera such as *Agaricus* and *Cryptococcus*, which reflect ecological adaptations for aerial dispersal. These patterns illustrate remarkable fungal niche specialisation and underscore the power of molecular methods in revealing biodiversity overlooked by culture-based approaches.

#### 3.3.3. Functional and genomic insights from extreme environments

Metagenomic studies of Arctic permafrost have identified *Rhodotorula frigidialcoholis* (formerly, *R*. JG1b) as the dominant psychrotolerant yeast. This species expresses cold-adapted lipases and proteases and metabolises ethanol even at subzero temperatures, making it a promising candidate for bioremediation in polar regions. Functional metagenomic annotation confirms novel metabolic pathways that are aligned with environmental restoration applications (Fox et al. 2022).

#### 3.3.4. Case study: post-fire fungal succession after the 2021 dixie fire

The 2021 Dixie Fire provided a unique opportunity to monitor post-disturbance soil fungal community dynamics. Systematic soil sampling was performed across burned and adjacent unburned plots, employing spatially replicated grids at varying distances from the fire perimeter. Environmental DNA was extracted using a standardised bead-beating technique. Fungal communities were profiled by amplifying the ITS region using ITS1F/ITS2 primers and sequenced on the Illumina MiSeq platform (Fox et al. 2022). Robust quality control included technical replicates and internal mock communities, with bioinformatic processing conducted in QIIME2 using the UNITE database. Rarefaction was applied to normalise the sequencing depth.

Multivariate analyses (Bray-Curtis dissimilarity and non-metric multidimensional scaling) demonstrated clear temporal fungal succession. During the first post-fire month, pyrophilous fungi, particularly *Pyronema* spp., dominated (~60%), supported by transcriptomic and metabolomic data indicating active decomposition and nutrient cycling (Tedersoo et al. 2014; Fox et al. 2022). Two years after the fire, mycorrhizal fungi such as *Glomus* (arbuscular) and *Rhizopogon* (ectomycorrhizal) accounted for approximately 60% of the sequences, facilitating soil stabilisation, plant recolonisation, and nutrient exchange network restoration, which are essential for ecosystem resilience.

(1) Cost-benefit and management implications: NGS enables a rapid and comprehensive assessment of pioneer and late-successional fungi, dramatically reducing the time and costs compared to traditional culturing or morphological identification. Insights from molecular data directly inform restoration planning by pinpointing the ideal timing and sites for seeding and mycorrhizal inoculation. Early identification of rising mycorrhizal populations has allowed local managers to coordinate restoration activities, with pilot projects reporting improved plant establishment and reduced soil erosion compared to standard protocols.

(2) Generalised relevance: This case exemplifies how high-throughput sequencing coupled with robust ecological and statistical analyses can streamline post-disturbance ecosystem management. It offers a scalable model for monitoring biodiversity and ecosystem health in fire-affected and other dynamic landscapes worldwide.

#### 3.3.5. Challenges in environmental molecular fungal surveys

Despite significant advances, several challenges persist.

- **Primer and database bias:** As detailed in Figure 2, commonly used primers (ITS1F/ITS2, 18S rRNA) substantially affect the apparent abundance of major fungal lineages, complicating comparisons across studies and potentially skewing ecological interpretations (Elbrecht and Leese 2015; Nilsson et al. 2019).
- **Viability discrimination:** Conventional eDNA cannot reliably distinguish viable, dormant, or relic fungal DNA. Emerging environmental RNA (eRNA) approaches show promise for identifying metabolically active fungi, but are not yet widely used (Baldrian et al. 2022).
- **Bioinformatic variation and annotation gaps:** Clustering methods (OTU/ASV) show analyst and platform dependency, leading to possible over- or underestimation of diversity. Reference databases are incomplete, and coverage for rare or poorly studied clades is noticeably lacking (Lindner and Banik 2011; Nilsson et al. 2019).

#### 3.3.6. Sector-wide synthesis and outlook

Environmental molecular mycology is now a cornerstone of real-time disturbance monitoring, ecosystem restoration, and global fungal surveillance, which are integral to One Health and biodiversity conservation frameworks. Unlocking its full potential requires sustained commitment to methodological standardisation, improved database curation, and technology transfer. Global initiatives such as UNITE, MycoBank, and the Global Biodiversity Information Facility (GBIF) exemplify collaborative progress. Case studies spanning wildfires, permafrost ecosystems, and extreme cliff habitats highlight both the achievements and ongoing constraints. The next major advances will hinge on integrating multi-omics, expanding eRNA methodologies, and fostering open, policy-supported, data sharing.

#### 3.3.7. Key takeaways for environmental mycology

- **Rich ecosystem insight:** eDNA metabarcoding and portable sequencing reveal comprehensive fungal diversity, functional roles, and ecological succession, especially following environmental disturbances.
- **Restoration and conservation:** Molecular data increasingly guide land management, optimising restoration timing, mycorrhizal inoculation, and erosion control to enhance ecosystem recovery.
- **Global surveillance:** Advances in portable, real-time sequencing democratise ecosystem monitoring, extending access to a broad community, including researchers, conservationists, and citizen scientists.
- **Best practices:** Environmental practitioners should prioritise integration with global fungal databases, invest in accessible open-source bioinformatics platforms, and adopt emerging environmental RNA approaches to improve the detection of active fungal communities.

## 4. Current challenges and limitations

Despite significant progress in molecular fungal diagnostics, substantial challenges persist that impact accuracy, reproducibility, and worldwide accessibility. For example, molecular barcoding—while promising—continues to face limitations due to an insufficient number of reliable, distinctive genetic markers (Gherbawy and Voigt 2010). Additionally, high variability within species in conventional molecular characters complicates the clear delineation of species boundaries, which further restricts taxonomic resolution (Gherbawy and Voigt 2010).

Addressing these barriers remains essential for realising the full potential of molecular approaches across clinical, agricultural, and environmental applications. Table 5 summarises these challenges alongside proposed solutions, all substantiated by empirical evidence and recent literature (Balajee et al. 2009; Meyer et al. 2009; Miller and Tang 2009; Elbrecht and Leese 2015; Nilsson et al. 2019).

**Table 5.** Current challenges and proposed solutions in molecular fungal diagnostics.

Category	Recommended protocols/platforms	Purpose/notes	References
DNA extraction	CTAB method, DNeasy Plant Mini Kit	High-yield DNA isolation from diverse fungal samples	Miller and Tang (2009)
Bioinformatics pipelines	QIIME2, Galaxy	Standardized, reproducible analysis workflows	Afgan et al. (2018); Bolyen et al. (2019)
ITS subregion selection	UNITE guidelines (ITS1/ITS2)	Consistent primer selection for metabarcoding studies	Nilsson et al. (2019)
Antifungal susceptibility testing	CLSI MM18-A, M27-A3, M38-A2	Standardized protocols for resistance profiling	Miller and Tang (2009); CLSI (2022)
Database curation	UNITE, MycoBank	Taxonomic validation, sequence annotation	Nilsson et al. (2019); Kõljalg et al. (2020)

### 4.1. Database inaccuracies and annotation errors

#### 4.1.1. Nature and causes of database errors

Public repositories such as GenBank and EMBL-EBI contain a significant proportion of misannotated fungal sequences—up to 10%–20% at the species level (Nilsson et al. 2019). These errors originate from incomplete taxonomic references, poor-quality or missing metadata, and historical use of outdated classifications. For instance, frequent misidentification occurs within the *Aspergillus niger* and *Candida* complexes due to morphological similarities and legacy data (Geiser et al. 1998; Balajee et al. 2009).

#### 4.1.2. Clinical and agricultural consequences

Annotation errors can have major real-world impacts. In clinical settings, misidentification of *Candida* species (e.g., confusing *C. auris* with *C. haemulonii* or *C. parapsilosis*) can directly result in inappropriate antifungal therapy, prolonged illness, increased healthcare costs, or uncontrolled outbreaks—especially since some species such as *C. auris* and *C. glabrata* can be resistant to commonly used drugs like fluconazole (Brown et al. 2012; Lockhart et al. 2017). In agriculture, inaccurate identification of mycotoxin-producing taxa (e.g., *Aspergillus flavus*) can lead to missed intervention windows and unmitigated crop losses (Gherbawy et al. 2015).

#### 4.1.3. Harmonisation and curation strategies for global surveillance

To mitigate these risks and enable global fungal surveillance, leading curated databases such as UNITE, MycoBank, and others employ rigorous curation and cross-database collaboration:

- **Voucher linking:** Each sequence must be linked to a physical voucher specimen—such as a herbarium or culture collection sample—ensuring traceability and enabling future taxonomic re-evaluation.
- **Peer-reviewed taxonomic placement:** Reference sequences are only accepted after peer-reviewed confirmation by taxonomic experts.
- **Standardised metadata:** Submissions require detailed, standardised metadata (e.g., sampling location, substrate, host, collection method), facilitating interoperability.
- **Taxon hypothesis (TH) framework:** UNITE employs technical clustering (TH IDs) of ITS sequences, accommodating both named and so-called “dark taxa”. Regular community-driven curation and expert re-examination continually update ambiguous or conflicting annotations (Nilsson et al. 2019; Kõljalg et al. 2020).
- **Quality control pipelines:** Sequences undergo chimera checking, quality trimming, and clustering using tools such as VSEARCH and QIIME2.
- **Community annotation and recurring updates:** Ambiguous taxa are flagged for community review and, if necessary, reclassified with each new database release.
- **AI-assisted annotation:** Tools like the RDP Classifier provide automated first-pass taxonomy, flagging possible errors by cross-referencing curated databases (Wang et al. 2007).

#### 4.1.4. Examples of successful harmonisation

A recent clinical survey found that up to 15% of *Candida* isolates would have been misidentified using uncurated GenBank data; however, harmonized identification through UNITE’s curated taxonomy greatly improved accuracy and patient outcomes (Lockhart et al. 2017; Nilsson et al. 2019). Similarly, UNEP and FAO agricultural surveillance programmes now rely heavily on curated datasets to monitor *Aspergillus* species, enabling more rapid international response against food safety threats.

#### 4.1.5. Implications for reliable diagnostics

Adoption of harmonised standards—including voucher linkage, expert community review, standardised metadata, deployment of operational TH models, AI-assisted annotation, and periodic curated updates—remains critical for trustworthy molecular fungal diagnostics and efficient global surveillance. Laboratories and researchers are urged to utilise only such rigorously curated, interoperable sources to maximise diagnostic reliability and reproducibility.

Table 6 summarises UNITE’s operational guidelines, metadata standards, TH assignment, and curation protocols (Nilsson et al. 2019; Kõljalg et al. 2020).

**Table 6.** Recommended protocols and platforms for standardisation in fungal molecular diagnostics.

Challenge	Description	Impact	Proposed solutions	References
Database inaccuracies	Misannotated sequences in public repositories (e.g., GenBank)	Misidentification of species; low diagnostic confidence	Curated databases (UNITE, MycoBank); community-driven annotation	Schoch et al. (2012); Nilsson et al. (2019); CLSI (2022)
Primer bias	Unequal amplification of taxa during PCR/metabarcoding	Skewed biodiversity estimates; false negatives/positives	Improved primer design (e.g., degenerate primers); *in silico* validation tools	Ihrmark et al. (2012); Elbrecht and Leese (2015); Tedersoo et al. (2015)
Cryptic speciation	Morphologically indistinguishable but genetically distinct taxa	Diagnostic errors; incorrect treatment decisions	Multilocus sequencing (TEF1-α, β-tubulin); whole-genome SNP analysis	Gherbawy et al. (2015); O’Donnell et al. (2015); Rhodes et al. (2017)
Standardization gaps	Lack of uniform protocols for extraction, amplification, and analysis	Poor reproducibility across labs; incompatible datasets	CLSI/ISO guidelines; UNITE consortium protocols for ITS sequencing	Schoch et al. (2014); Nilsson et al. (2019); CLSI (2022)
Cost and accessibility	Expensive instruments/reagents; limited infrastructure in low-resource settings	Limited adoption in field clinics or developing regions	Portable devices (e.g., MinION); democratized platforms (e.g., CRISPR-based assays)	Chen et al. (2018); WHO (2022); Weilguny et al. (2023)

### 4.2. Primer bias and amplification artifacts

#### 4.2.1. Primer bias in PCR

Primer bias continues to pose a significant challenge in PCR-based assays, often resulting in an unbalanced representation of fungal communities within metabarcoding studies. For example, the commonly used ITS1F/ITS2 primer pair amplifies Ascomycota approximately 1.5 to 2 times more efficiently than Basidiomycota, leading to distorted biodiversity estimates in forest soils (Tedersoo et al. 2015). Likewise, computational analyses have demonstrated that 18S rRNA primers preferentially amplify Chytridiomycota, while underrepresenting Glomeromycota by as much as 40% (Elbrecht and Leese 2015).

#### 4.2.2. Strategies to counteract bias

Several strategies have been developed to address these biases:

- The use of degenerate primers and blocking oligonucleotides has improved the coverage of underrepresented taxa. For example, the ITS3/ITS4NG primers have been shown to mitigate bias against *Glomeromycota* (Ihrmark et al. 2012).
- Additionally, in silico validation tools such as Primer Prospector facilitate the optimisation of primer design by predicting binding efficiency across a broad range of fungal lineages (Mbareche et al. 2021).

#### 4.2.3. Ongoing challenges

Overall, while primer bias remains a persistent issue, ongoing methodological advances are contributing to more accurate assessments of fungal diversity.

### 4.3. Cryptic speciation and intraspecific variation

#### 4.3.1. Challenges in species delimitation

Cryptic species complexes—groups of fungi that are morphologically indistinguishable yet genetically distinct—represent a fundamental challenge in fungal diagnostics and taxonomy. Such complexes occur in clinically and agriculturally important lineages, including the *Candida parapsilosis* group and *Aspergillus flavus* species complex, where overlapping morphological traits and limited genetic divergence in standard barcode regions hinder accurate identification. Compounding this issue, intragenomic variation within commonly used markers such as the Internal Transcribed Spacer (ITS) region can be substantial; for example, *Armillaria gallica* exhibits up to 4% ITS sequence variability within individual genomes, a divergence level typically expected between separate species (Lindner and Banik 2011). This intragenomic heterogeneity complicates operational species boundaries and increases the risk of misclassification.

#### 4.3.2. Molecular solutions

To overcome these limitations, multilocus sequencing incorporating additional phylogenetically informative markers—such as β-tubulin, RNA polymerase II subunit (RPB2), and translation elongation factor 1-alpha (TEF1-α)—has become instrumental in disentangling cryptic lineages. These protein-coding genes provide increased resolution due to higher mutation rates and reduced homoplasy, enabling more precise species delimitation within complexes like *Fusarium* (O’Donnell et al. 2015).

Furthermore, advances in whole-genome sequencing (WGS) and genome-wide single nucleotide polymorphism (SNP) analysis have ushered in a new era of genome-based taxonomy, dramatically enhancing taxonomic resolution beyond single- or multilocus approaches. Genome-scale data enable the differentiation of strains and cryptic species complexes with unparalleled precision, as exemplified by studies on *Cryptococcus neoformans/gattii* complexes crucial for outbreak tracking and epidemiological control (Rhodes et al. 2017). Through comprehensive genome comparisons, researchers can identify fine-scale genetic differences reflecting evolutionary divergence, pathogenicity traits, and antifungal resistance profiles, thus enabling robust and reproducible species boundaries.

#### 4.3.3. Transformative potential

Collectively, integrating multilocus sequencing with genome-based taxonomy constitutes a transformative approach to resolving cryptic speciation, reducing diagnostic ambiguity, and improving the accuracy and reliability of fungal identification critical for clinical management, agriculture, and biodiversity assessment.

### 4.4. Standardisation and reproducibility gaps

#### 4.4.1. Variability across protocols

There is considerable heterogeneity in laboratory protocols across the field, spanning DNA extraction methods (such as bead-beating versus enzymatic lysis) and bioinformatics workflows (e.g., QIIME2 vs. mothur), which complicates the comparability of studies. A 2018 meta-analysis demonstrated that the selection of ITS subregions alone (ITS1 versus ITS2) could account for approximately 30% of the observed variability in soil fungal community profiles (Sommermann et al. 2018).

#### 4.4.2. Efforts toward harmonisation

Efforts to enhance standardisation and reproducibility include the adoption of CLSI (2022) guidelines (such as MM18-A) for antifungal susceptibility testing and the use of unified protocols for ITS analysis provided by the UNITE consortium (Miller and Tang 2009). Additionally, open-source bioinformatics platforms like Galaxy have streamlined analytical workflows and improved reproducibility across research institutions (Afgan et al. 2018). Table 6 summarises recommended protocols and platforms to address these challenges, emphasising standardised methodologies for DNA extraction, bioinformatics, and database curation.

### 4.5. Cost, accessibility, and infrastructure disparities

#### 4.5.1. Inequities in global diagnostic capacity

Advanced molecular diagnostic platforms remain inaccessible to many laboratories in low-resource regions, despite the high burden of invasive fungal infections and crop pathogens in these areas. For instance, whole-genome sequencing typically costs around $500 per sample and requires infrastructure that is often lacking in regions such as sub-Saharan Africa, limiting participation in global surveillance initiatives (Meyer et al. 2009).

#### 4.5.2. Emerging solutions and initiatives

Potential solutions include the deployment of portable sequencing devices, such as the Oxford Nanopore MinION, which facilitate real-time diagnostics in the field at a relatively accessible cost (under $1,000 per device) (Ho et al. 2020). Furthermore, public-private initiatives like the WHO’s Fungal Priority Pathogens List are supporting technology transfer and training programmes in underserved areas (WHO 2022).

## 5. Future directions

The field of molecular fungal diagnostics is advancing rapidly, providing unprecedented opportunities to overcome longstanding challenges and expand fungal identification in clinical, agricultural, and environmental settings. Central to these advances are growing genomic databases and genetic marker libraries that underpin molecular barcoding, continually refined by bioinformatics methods that reveal the immense fungal diversity (Gherbawy and Voigt 2010). Despite this, issues such as database inaccuracies, primer biases, and unequal access to advanced technologies in under-resourced regions remain obstacles. Contemporary progress in integrative taxonomy, artificial intelligence (AI), portable sequencing, and international collaborations is beginning to address these gaps. Table 5 details prevailing challenges and promising solutions (Miller and Tang 2009; Elbrecht and Leese 2015; Nilsson et al. 2019). Table 5 outlines the current obstacles alongside promising emerging solutions (Miller and Tang 2009; Elbrecht and Leese 2015; Nilsson et al. 2019).

### 5.1. Integrative taxonomy

Fungal taxonomy now leverages integrative multi-omics methods. Traditional classification based on morphology or single-gene sequencing often fails to capture fungi’s functional and genetic complexity. Whole-genome sequencing (WGS), together with metabolomic and transcriptomic assessments, provides deeper biological insights (Lücking et al. 2020). High-throughput phenotyping connects genotype to observable traits, directly informing clinical diagnostics and agricultural breeding.

**Clinical example:** Multi-omics unravel the gene regulatory networks behind azole resistance and virulence in *Aspergillus fumigatus*, informing targeted treatment (Bottery et al. 2024).

**Agricultural example:** Transcriptomic and proteomic profiling in *Aspergillus flavus* reveals genes linked to mycotoxin production and maize resistance, aiding breeding for disease resistance (Liu et al. 2021).

**Environmental example:** multi-omics identify functional shifts in fungal communities under environmental stresses such as drought or pollution, enhancing ecosystem resilience strategies.

### 5.2. Artificial intelligence and machine learning

AI and machine learning integration is vital to manage and analyse massive sequencing and imaging datasets. Algorithms enable automated annotation, resistance mutation prediction, and discovery of cryptic or novel species by detecting subtle patterns invisible to manual review (Ahrendt et al. 2018).

**Clinical example:** Deep learning classifies fungal ITS sequences with > 95% accuracy and predicts azole resistance mutations in *Aspergillus fumigatus*, improving clinical outcomes (Perlin et al. 2017).

**Agricultural example:** AI-powered smartphone apps monitor crop leaves to detect early fungal disease symptoms, assisting in timely disease management.

**Environmental surveillance:** AI analyzes environmental DNA (eDNA) to detect invasive fungi and biodiversity changes, supporting early ecosystem management interventions.

As datasets grow, AI’s role in fungal diagnostics, surveillance, and outbreak forecasting will expand.

### 5.3. Sequencing technologies become widely available

Portable sequencing technologies such as the Oxford Nanopore MinION have democratised fungal diagnostics, enabling on-site, real-time identification in resource-limited or remote areas. Removal of traditional infrastructure barriers facilitates rapid response to outbreaks, agricultural diseases, and environmental monitoring, enhanced by cloud-based analytic platforms that enable broader participation from local labs to citizen scientists.

### 5.4. International data sharing and collaborative networks

Managing fungal threats efficiently requires robust international collaboration and open data exchange. Resources like UNITE and MycoBank standardise taxonomy and metadata, promoting consistency across nations. Coordinated surveillance networks reduce annotation errors and foster interoperability, underpinning integrated One Health approaches addressing clinical, agricultural, and environmental fungal threats collectively (Neves et al. 2023).

### 5.5. Precision mycology and personalised approaches

Innovations in molecular diagnostics enable precision mycology by linking pathogen genotypes with antifungal susceptibilities and clinical outcomes. This facilitates optimised treatments in healthcare and precision-targeted fungicide application or biocontrol in agriculture, reducing resistance emergence and crop losses. Continued reductions in sequencing and bioinformatics costs promise wider adoption of these approaches (Gilchrist et al. 2015; Perlin et al. 2017).

### 5.6. Future directions: multi-omics, environmental RNA, and public participation

Combining metagenomics, environmental transcriptomics (eRNA), and AI enhances detection of active fungal populations, outbreak prediction, and timely intervention strategies. Multi-omics also clarifies species boundaries, gene flow, and fungal community functions under environmental stresses. Lowered costs and user-friendly tools facilitate adoption by agricultural extension services and informed public participation, expanding global surveillance and fungal threat response capacity.

### 5.7. Future-focused practical implications across sectors

Advances point to transformative trends in the next 5 years:

- Increasing reliance on AI for rapid, computerised diagnostics and real-time resistance and outbreak detection across clinical, agricultural, and environmental contexts.
- Democratisation of sequencing through portable, cloud-enabled devices enabling global access, including underserved and remote areas.
- Enhanced precision in mycology from integrative genotypic, phenotypic, and multi-omics data that allow unprecedented, targeted interventions.
- The critical role of international cooperation, standardised data sharing, protocols, and workforce training as foundations for effective global fungal surveillance within a One Health framework.

Professionals will experience dramatically reduced diagnostic turnaround; expanded field-deployable molecular tools; improved diagnosis accuracy driven by AI; and stronger integration of diagnostics with control and management efforts, leading to improved health, food security, and ecosystem resilience.

## 6. Conclusions

The landscape of fungal diagnostics has undergone a seismic transformation in recent years, largely driven by molecular technologies. DNA barcoding, multilocus sequencing, and next-generation sequencing have collectively redefined our ability to detect and characterise fungal species across clinical, agricultural, and environmental contexts. Such methods now facilitate rapid, highly accurate identification and have vastly improved pathogen surveillance and ecological monitoring. Yet, it would be misguided to disregard traditional morphological and culture-based methods. These approaches remain essential, particularly when working with well-characterised species or in settings where molecular infrastructure is lacking. In many cases, the basics—microscopy, petri dishes, classic culturing—are still the mainstay of diagnostics, especially in resource-limited environments.

Recent technological innovations, including CRISPR-based assays and portable LAMP platforms, have further democratised access to advanced diagnostics. For instance, CRISPR technology now enables on-site detection of pathogens such as *Phakopsora pachyrhizi* in asymptomatic soybean plants, and LAMP assays have drastically reduced diagnostic turnaround times for *Fusarium graminearum* in wheat. These advances are game-changing for agricultural systems where rapid response is critical for food security. Nevertheless, significant challenges persist. Discriminating pathogenic from commensal fungal taxa in mycobiome studies remains complex, complicated by database errors, primer biases, and intragenomic ITS variability. Additionally, disparities in access to advanced diagnostics continue to hinder progress in low-resource regions. Overcoming these obstacles will require adoption of standardised protocols, such as ISO 17025, robust genomic databases like UNITE, and sustained investment in both infrastructure and workforce development.

Looking forward, integrating multi-omics data with computational and machine learning approaches holds considerable promise. Such strategies could usher in a new era of precision mycology, enabling interventions tailored to individual patients or to specific agricultural or environmental contexts. In an age of climate change, antimicrobial resistance, and increasing global interconnectedness, molecular diagnostics represent indispensable tools for protecting public health, enhancing food security, and conserving ecosystems. Ultimately, successful adoption of these technologies will depend on interdisciplinary collaboration within a One Health framework. Ensuring equitable access and fostering innovation across clinical, agricultural, and environmental domains will be crucial to maximising the benefits of molecular diagnostics for diverse communities worldwide.
